# Microstructure and Texture Variations in High Temperature Titanium Alloy Ti65 Sheets with Different Rolling Modes and Heat Treatments

**DOI:** 10.3390/ma13112466

**Published:** 2020-05-28

**Authors:** Ding Zhao, Jiangkun Fan, Zhixin Zhang, Xudong Liu, Qingjiang Wang, Zhiyong Chen, Bin Tang, Hongchao Kou, Shuanxiao Jia, Jinshan Li

**Affiliations:** 1 State Key Laboratory of Solidification Processing, Northwestern Polytechnical University, Xi’an 710072, China; zhaoding@mail.nwpu.edu.cn (D.Z.); jerry888526@126.com (Z.Z.); liu_xudong666@163.com (X.L.); toby@nwpu.edu.cn (B.T.); hchkou@nwpu.edu.cn (H.K.); ljsh@nwpu.edu.cn (J.L.); 2 National & Local Joint Engineering Research Center for Precision Thermoforming Technology of Advanced Metal Materials, Xi’an 710072, China; 3 Baoti Group Ltd., Baoji 721014, China; 4 Institute of Metal Research, Chinese Academy of Sciences, Shenyang 110016, China; qjwang@imr.ac.cn (Q.W.); zychenzychen@163.com (Z.C.); 5 Baoji Titanium Industry Co., Ltd., Baoji 721014, China; jiashuanxiao@baoti.com

**Keywords:** high temperature titanium alloy, rolling mode, microstructure, texture, recrystallization, variant selection

## Abstract

Ti65 alloy (Ti-5.8Al-4.0Sn-3.5Zr-0.5Mo-0.4Si-0.3Nb-1.0Ta-0.8W-0.05C) is the newly developed high temperature titanium alloy optimized from Ti60 alloys. The long-term service temperature of the alloy is as high as 650 °C, which is unattainable with the previous high temperature titanium alloy. It has excellent strength and excellent creep resistance, and has great application prospects in the aerospace industry. In the current study, the evolution of microstructure and texture of Ti65 alloy sheets developed by unidirectional rolling (UDR) and cross rolling (CR) followed by solution and aging treatment was investigated. The microstructure of the UDR sample consists of equiaxed $\alpha_{p}$, lamellar $\alpha_{s}$ and few elongated $\alpha_{p}$, and the texture is the combination of minor B-type and major T-type texture, with the main component of basal {0001} fiber texture and $\left\{01\bar{1}0\right\}$$<2\bar{1}\bar{1}0>$, respectively. Due to more active slip system resulted by transformed direction, the microstructure of the CR sample consists of more elongated $\alpha_{p}$, and the $\left\{01\bar{1}0\right\}$<0001> texture characterized as R-type texture forms in addition to B/T-type texture. With aging temperature increasing, the microstructures for both transform to duplex microstructure, and the thicknesses of lamellar $\alpha_{s}$ increase. B-type texture becomes stronger, while T/R-type texture are weakened, which is caused by the combination of recrystallization, spheroidization, and variant selection. An abnormal increasing of T/R-type texture but constant B-type texture happens in the CR-600 sample, which is related to high recrystallization fraction. It is expected that the research results can provide useful references for the rolling of high temperature titanium alloy sheets and the precise control of microstructure/texture.

## 1. Introduction

High temperature titanium alloys (such as IMI834, Ti-1100, Ti6242, etc.) play a significant role in the aviation and aerospace industry due to low density, high strength, excellent corrosion resistance, and high temperature resistance at service temperature [1,2,3]. To satisfy the application requirements, titanium alloy sheets are manufactured by large deformation like rolling, and hence microstructure and texture can change drastically, which in turns affects the mechanical properties and its anisotropy [4,5]. For instance, due to the hexagonal close-packed (HCP) crystal structure of α phase and the c/a ratio of Ti (1.587), slip is the principal mode during α + β phase region deformation, with the principal slip system of prismatic $\left\{10\bar{1}0\right\}\left(11\bar{2}0\right)$ and the secondary slip system of basal $\left\{0001\right\}\left(11\bar{2}0\right)$ [6]. Hence, strong basal texture (B-type texture), i.e., grains with {0001} planes lying nearly parallel to the plane of the sheet, and transverse texture (T-type texture), i.e., grains with c axis being parallel or close to transverse direction, exist in unidirectional hot rolled titanium alloy sheets.

Ti65 alloy (Ti-5.8Al-4.0Sn-3.5Zr-0.5Mo-0.4Si-0.3Nb-1.0Ta-0.8W-0.05C) is the newly developed near α titanium alloy optimized from Ti60 alloy [7]. The long-term service temperature of the alloy is as high as 650 °C, which is unattainable with the previous high-temperature titanium alloy. It has excellent strength and excellent creep resistance, and has great application prospects in the aerospace industry. In general, due to smaller number of active slip system in α phase [8], Ti65 alloy sheets are manufactured by hot deformation in the α + β phase field in order to reduce deformation resistance, and followed by solution plus aging treatment to obtain excellent mechanical properties. As is widely known, rolling processing parameters, like rolling mode and heat treatment, can strongly influence the microstructure and texture of sheets, and in turn mechanical properties. It is reported that Gupta et al. [9] performed unidirectional rolling and multi-step cross rolling on Ti-15V-3Cr-3Sn-3Al alloys, respectively. For the unidirectional rolling sheet, strong α and γ-fibers of β phase were observed, while strong rotated cube ({100}〈110〉) texture developed during multi-step cross rolling. Nilesh et al. [10] studied texture evolution during unidirectional rolling and multi-step cross rolling. A weak and discontinuous rolling γ fiber texture of β phase was observed in the unidirectional rolling sheet, while it became stronger and continuous in the multi-step cross rolling sheet, which is attributed to the homogeneous deformation taking place during multi-step cross rolling.

Heat treatment is an efficient way to adjust microstructure characteristics and improve mechanical properties of titanium alloys [11,12], and it has been studied extensively. For instance, Bhattacharyya et al. [13] proved that the initial rolling texture of α phase of Ti-6Al-4V alloy showed a strengthening after heat treatment at 800 °C, which was attributed to recrystallization of α grains. Roy et al. [14] reported that the spread of fibers caused by spheroidization of $\alpha_{p}$ phase occurred on annealed Ti-6Al-4V alloy. Manda et al. [15] aged Ti-5Al-5Mo-5V-3Cr alloy samples at different temperature from low to high. The α phase texture in low and high temperature are strong c-type basal and prismatic texture respectively, which may be resulted from variant selection that affected volume fraction of β phase. The transformation process of α→β→α during heat treatment is most typical and important in titanium alloys. According to Burgers orientation relationship [16], i.e., $\{110{\}}_{\beta }∥\{0001{\}}_{\alpha }\;\mathrm{and}\;<111{>}_{\beta }∥<11\bar{2}0{>}_{\alpha }$, and symmetry of the crystal, a single α orientation can give rise to six equivalent β orientations and a single β orientation can generate up to 12 equivalent α orientations with equal probability, respectively [17,18,19]. As a result, a single $\alpha_{p}$ orientation can result in 72 $\alpha_{s}$ orientations, while 12 of the combinations will restore the original α phase [20], and hence rolling texture should have been weakened or even disappeared during α→β→α phase transformation, if without variant selection. Current research has shown the existence of variant selection during phase transformation, and it is affected by several factors, such as deformation temperature, extent of deformation, mode of deformation, and the most important, which is heat treatment [21].

The evolution of microstructure and texture during rolling and heat treatment affects mechanical properties and its anisotropy. For instance, Ghosh studied the effects of rolling mode on the evolution of mechanical properties of commercial pure titanium [22]. The micro-hardness values measured of unidirectional rolled sheet are higher in comparison to multistep cross rolled sheet whether in as-rolled condition or annealed condition. Wang reported the low anisotropy of mechanical properties in commercial pure titanium sheets rolled by cross rolling process in comparison to sheets rolled by the unidirectional rolling process [23]. According to the research of Li [24], the ductility of TC6 alloy increases with increasing aging temperature and time, but the tensile strength changes nearly nothing or even decreases. Hence, the microstructure and texture evolution mechanisms need to be paid much attention, which can provide theoretical support for the sheet rolling deformation of high temperature titanium alloys.

However, as the newly developed high temperature titanium alloy, only a few studies of the Ti65 alloy were taken [25,26,27]. To clarify the evolution mechanism of microstructure and texture and so on achieving precise control of microstructure and texture, the systematic studies of Ti65 alloy during hot deformation and subsequent heat treatment are necessary. In the present study, Ti65 alloy sheets are obtained by two different rolling modes, namely unidirectional rolling (UDR) and cross rolling (CR), followed by solution and subsequent aging treatment. The optical microscope (OM), scanning electron microscope (SEM), X-ray diffraction (XRD), and electron backscatter diffraction (EBSD) techniques were used to systematically characterize and analyze the microstructure and texture characteristics of the alloy sheets. These studies will further promote the understanding of the effects of rolling methods and heat treatments processes on the microstructure and texture evolution of Ti65 alloy sheets, and provide technical guidance for the actual processing and production of high temperature titanium alloy sheets.

## 2. Material and Experimental Procedure

**Figure 1:**
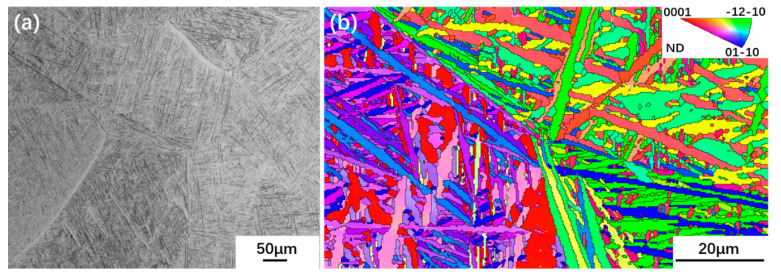
Microstructure (**a**) and inverse pole figure (IPF) map (**b**) of the as-received Ti65 alloy billet.

**Figure 2:**
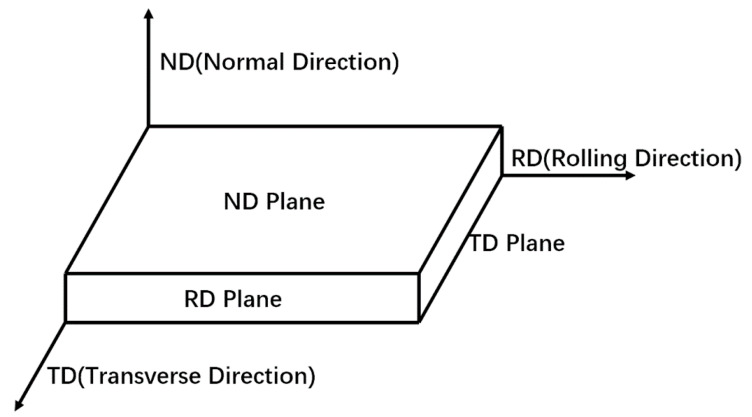
Three-dimensional representation of RD, TD, and ND planes in the rolled sheet.

The as-received Ti65 alloy billet with a thickness of 18 mm was supplied by Baoti Group Ltd. (Baoji, China), and the β transus temperature determined by optical microscopy is 1035 °C. The as-received billet was obtained by solution treatment at 1070 °C for 30 min followed by water quenching, and hence the microstructure of the as-received billet shown in Figure 1a was composed of acicular martensite α′ with lamellar of 2 µm in width bestrewing in the β grains. The inverse pole figure (IPF) map shown in Figure 1b indicates the orientations of α′ grains in β grain are different from that in adjacent β grain, while the orientations in one β grain are inclined to be close or even parallel. Ti65 alloy sheets with a thickness of 2 mm was manufactured from the as-received billet by UDR and CR modes, with the thickness deformation of 50% imparted to each step, as shown in Table 1. Samples with dimension of 15 mm × 10 mm machined from two hot rolled sheets were subjected to solution treatment at 990 °C for 30 min followed by air cooling (AC), and subsequently aged at 600 °C and 700 °C for 5 h, respectively, and then cooled in the air. To facilitate discussion in the following text, three-dimensional representations of RD (rolling direction), TD (transverse direction), and ND (normal direction) planes in the rolled sheet are shown in Figure 2. All conventions used for RD and TD refer to the RD and TD of the UDR sheet.

The metallographic examination samples were prepared through mechanical grinding, polishing, and subsequently etched with an etching solution with the volume ratio of HF:HNO_3_:H_2_O = 1:2:50. Microstructure of etched samples in RD plane and TD plane was examined by using OLYMPUS optical microscope (DP71, Olympus Corporation, Tokyo, Japan) and FEI Nova field emission scanning electron microscope (FEI, Hillsboro, OR, USA).

**Figure 3:**
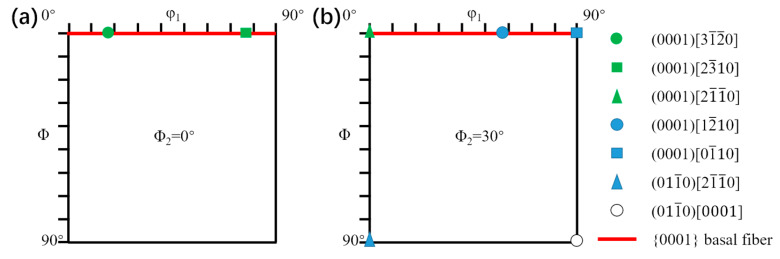
Standard orientation distribution function (ODF) maps of titanium alloys: (**a**) $\varphi_{2}$ = 0°; (**b**) $\varphi_{2}$ = 30°.

Texture measurements on ND plane of samples were carried out by using D8 Advance X-ray diffractometer (BRUKER, Karlsruhe, Germany). Because of less volume fraction of β phase in Ti65 alloy at room temperature, it can be conferred the measured texture belongs to α phase. For XRD, three pole figures (0001), ($11\bar{2}0$), and ($10\bar{1}0$) of α phase were measured in a 5° by 5° grid. Complete orientation distribution function (ODF) maps were calculated based on obtained pole figures by HKL Channel 5 software. Due to the symmetry of HCP crystal structure, only the ODF plots of constant Φ and $\varphi_{1}$ section with $\varphi_{2}$ = 0° and $\varphi_{2}$ = 30° in Euler space defined by three Euler angles, i.e., $\varphi_{1}$, Φ and $\varphi_{2}$, are presented. The standard $\varphi_{2}$ = 0° and $\varphi_{2}$ = 30° ODF maps calculated by Equation (1) [28] are shown in Figure 3a,b respectively, where (HKIL)[UVTW] are miller indices and {φ_1_Φφ_2_} are Euler angles.

(1) $$\begin{array}{c} \left[\begin{array}{c} \begin{array}{c} H\\ K \end{array}\\ I\\ L \end{array}\right]=\left[\begin{array}{ccc} \sqrt{3}/2 & -1/2 & 0\\ 0 & 1 & 0\\ \begin{array}{c} -\sqrt{3}/2\\ 0 \end{array} & \begin{array}{c} -1/2\\ 0 \end{array} & \begin{array}{c} 0\\ c/a \end{array} \end{array}\right]\cdot \left[\begin{array}{c} {\sin\Phi \sin\varphi }_{2}\\ {\sin\Phi \cos\varphi }_{2}\\ \cos\Phi \end{array}\right]\\ \left[\begin{array}{c} \begin{array}{c} U\\ V \end{array}\\ T\\ W \end{array}\right]=\left[\begin{array}{ccc} 1/\sqrt{3} & -1/3 & 0\\ 0 & 2/3 & 0\\ \begin{array}{c} -1/\sqrt{3}\\ 0 \end{array} & \begin{array}{c} -1/3\\ 0 \end{array} & \begin{array}{c} 0\\ a/c \end{array} \end{array}\right]\cdot \left[\begin{array}{c} -{\cos\Phi \sin\varphi }_{1}{\sin\varphi }_{2}{+\cos\varphi }_{1}{\cos\varphi }_{2}\\ -{\cos\Phi \cos\varphi }_{2}{\sin\varphi }_{1}-{\cos\varphi }_{1}{\sin\varphi }_{2}\\ {\sin\Phi \sin\varphi }_{1} \end{array}\right] \end{array}$$

Samples for EBSD analysis were prepared by electro-polishing ND plane in the solution of 5 mL perchloric acid, 35 mL Butyl Alcohol and 60 mL methanol at 35 V and 5 °C for 20–25 s. With Helios NanoLab G3 UC equipped with EBSD scanner (FEI, Hillsboro, OR, USA), a step size of 0.2–0.3 µm was performed on an area of 100 μm × 100 μm for all samples. The data obtained was processed with HKL Channel 5 software, and hence IPF maps, grain size maps, recrystallization maps, Kernel Average Misorientation (KAM) maps, and grain boundary misorientation maps were obtained. Grain boundaries with misorientation higher than 15° are denoted as high angle grain boundaries (HAGBs) and by thick black lines, and low angle grain boundaries (LAGBs) with misorientation lower than 15° are not shown.

The tensile specimens with the size of 40 mm × 55 mm and the gauge length of 20 mm are machined from the UDR sheet and the CR sheet along RD and TD direction, respectively. To facilitate discussion in the following text, the tensile specimens are named according to the direction along which they are machined. For instance, the sample machined from the UDR sheet along RD direction is called UDR-RD. The tensile tests are carried out by ETM105D universal testing machine (Test star-ETM105D, WANCE, Shenzhen, China) at room temperature, and the strain was measured by electronic extensometer. The data of average tensile strength (TS), average yielding strength (YS), and average elongation (EL) was obtained.

## 3. Results

### 3.1. Microstructure Characterization of Ti65 Alloy Sheets

**Figure 4:**
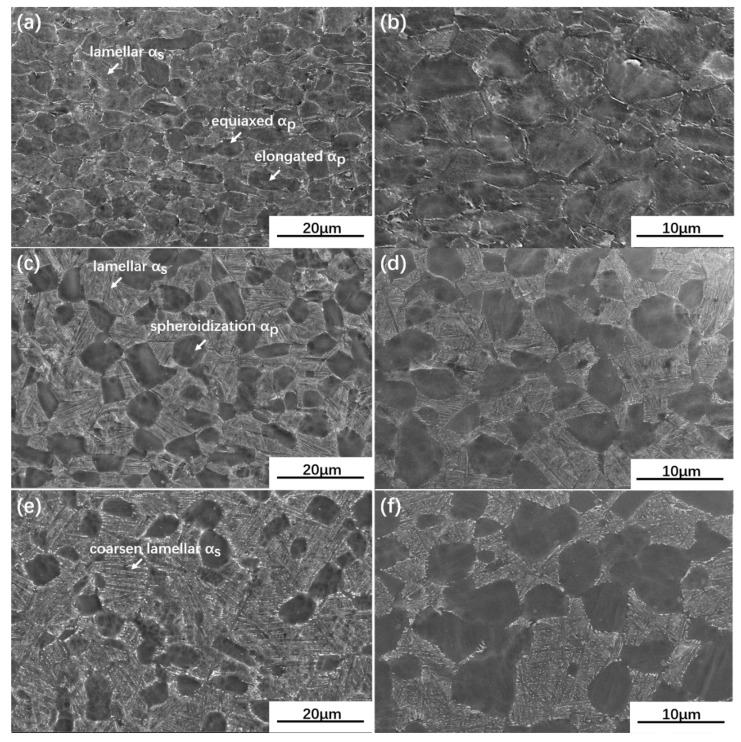
Scanning electron microscope (SEM) images of UDR Ti65 alloy sheets: (**a**,**b**) UDR; (**c**,**d**) UDR-600 and (**e**,**f**) UDR-700. (**a**,**c**,**e**) are SEM images of RD plane of samples of and (**b**,**d**,**f**) are of TD plane.

**Figure 5:**
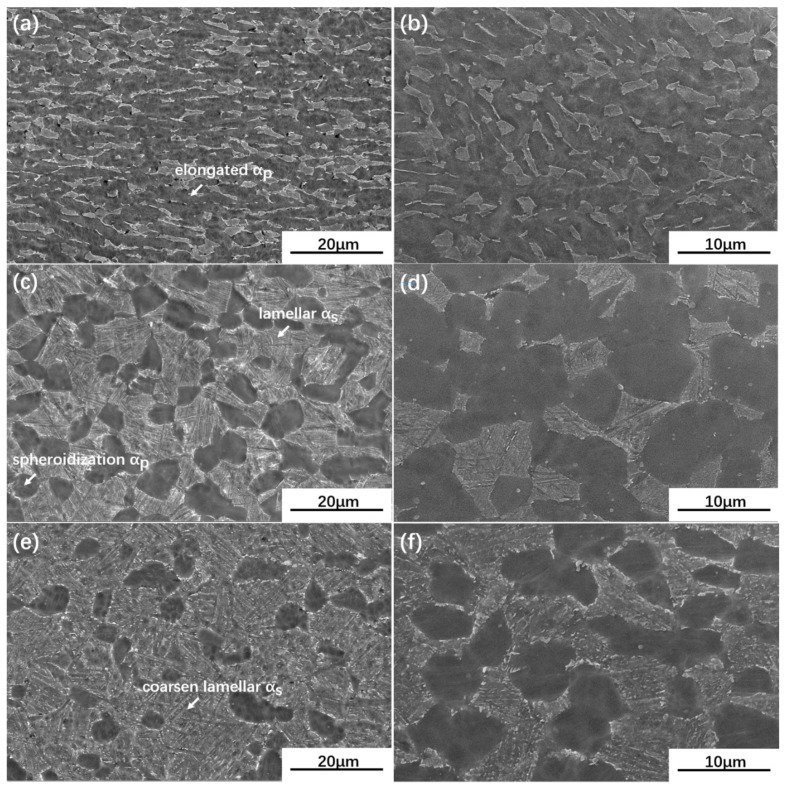
Scanning electron microscope (SEM) images of CR Ti65 alloy sheets: (**a**,**b**) CR; (**c**,**d**) CR-600; and (**e**,**f**) CR-700. (**a**,**c**,**e**) are SEM images of RD plane of samples of and (**b**,**d**,**f**) are of TD plane.

**Figure 6:**
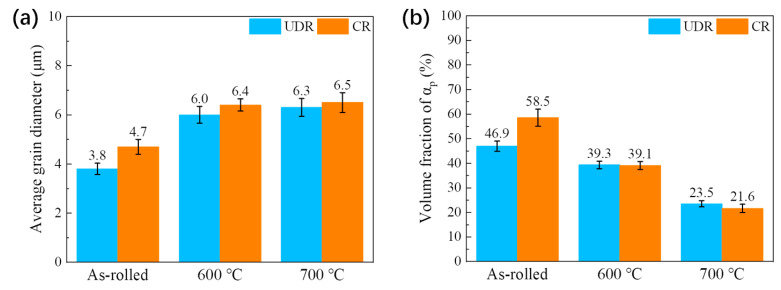
Average grain diameter diagrams (**a**) and volume fraction of $\alpha_{p}$ (**b**) of Ti65 alloy sheets.

The SEM images of UDR samples and CR samples are separately shown in Figure 4 and Figure 5. The images of the UDR sample reveal the presence of equiaxed structure consists of equiaxed $\alpha_{p}$, lamellar $\alpha_{s}$ and a few elongated $\alpha_{p}$, which are marked by an arrow. Simultaneously, elongated $\alpha_{p}$ grains rather than equiaxed $\alpha_{p}$ grains are dominant in the CR sample, which can be inferred from Figure 5a,b, indicates the benefit of CR process to homogeneous deformation, which may lead to less formation of cracks during hot rolling. The average α grain diameter of UDR samples shown in Figure 6a are finer than CR samples, and the volume fraction of $\alpha_{p}$ of UDR samples shown in Figure 6b are less. Compared to as-rolled samples, the microstructures of both UDR-600 and CR-600 samples transform to duplex microstructure, with spheroidized $\alpha_{p}$, lower volume fraction of $\alpha_{p}$ but higher average grain diameter. The average grain diameter of UDR and CR samples increases further with heat treatment temperature rising to 700 °C, but with lower difference. The volume fraction of $\alpha_{p}$ decreases, and more significant spheroidization response of $\alpha_{p}$ is observed. The formation rate of $\alpha_{s}$ during phase transformation is affected by diffusion rate of solute atoms [29], and hence increasing aging temperature can coarsen lamellar $\alpha_{s}$ by facilitating diffusion of solute atoms, which is in line with that the width of lamellar $\alpha_{s}$ increases from ~0.2 µm in the UDR-600 sample to ~0.4 µm in the UDR-700 sample and ~0.15 µm in the CR-600 sample to ~0.35 µm in the CR-700 sample.

**Figure 7:**
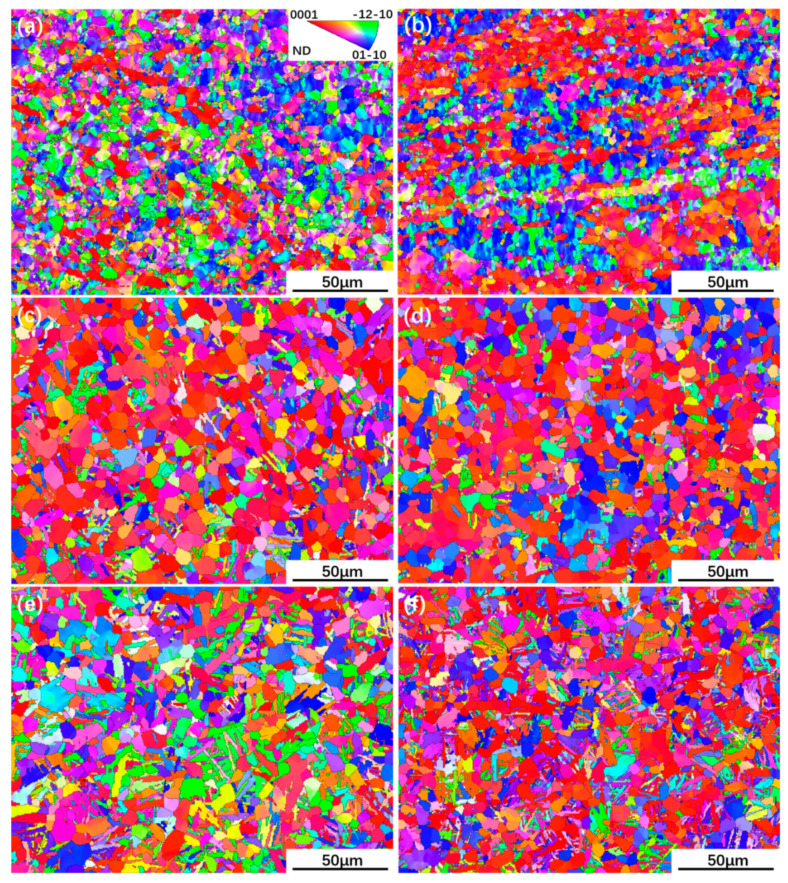
Electron backscatter diffraction (EBSD) IPF maps of Ti65 alloy sheets: (**a**) UDR; (**b**) CR; (**c**) UDR-600; (**d**) CR-600; (**e**) UDR-700; and (**f**) CR-700.

No obvious macro-zone in UDR and CR samples is observed, which can be conferred from Figure 7a,b, indicating that thermal deformation process can destroy macro-zone effectively [30]. The deformation modes realized by slip and twinning system are considered as the main cause of the texture evolution of metals. No deformation twinning is observed in both samples, which may be attributed to 5.8 wt.% Al content in Ti65 alloy and high rolling temperature [31]. Therefore, the slip mode has decisive effect on the texture evolution of Ti65 alloy sheets during hot rolling process [32].

**Figure 8:**
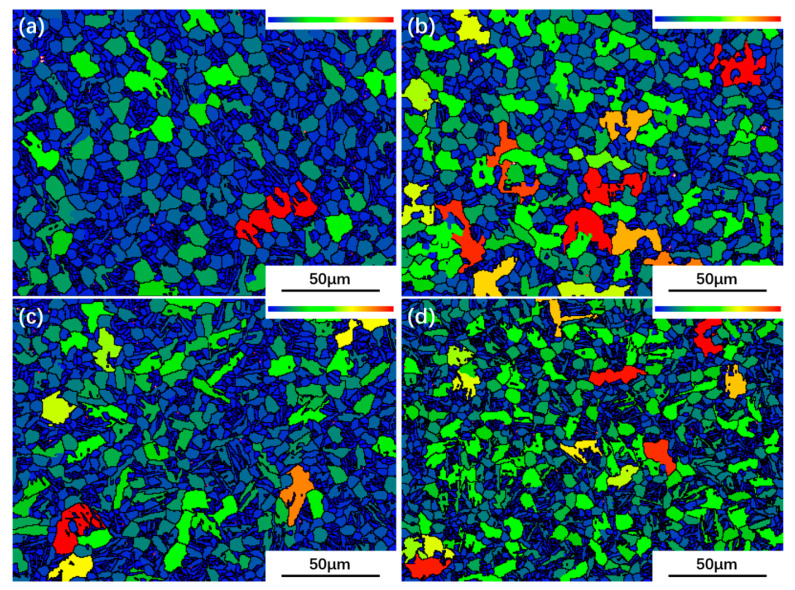
Maps of grain size of Ti65 alloy sheets: (**a**) UDR-600; (**b**) CR-600; (**c**) UDR-700 and (**d**) CR-700. Grains with maximum size are marked as red, and grains with minimum size as blue.

**Figure 9:**
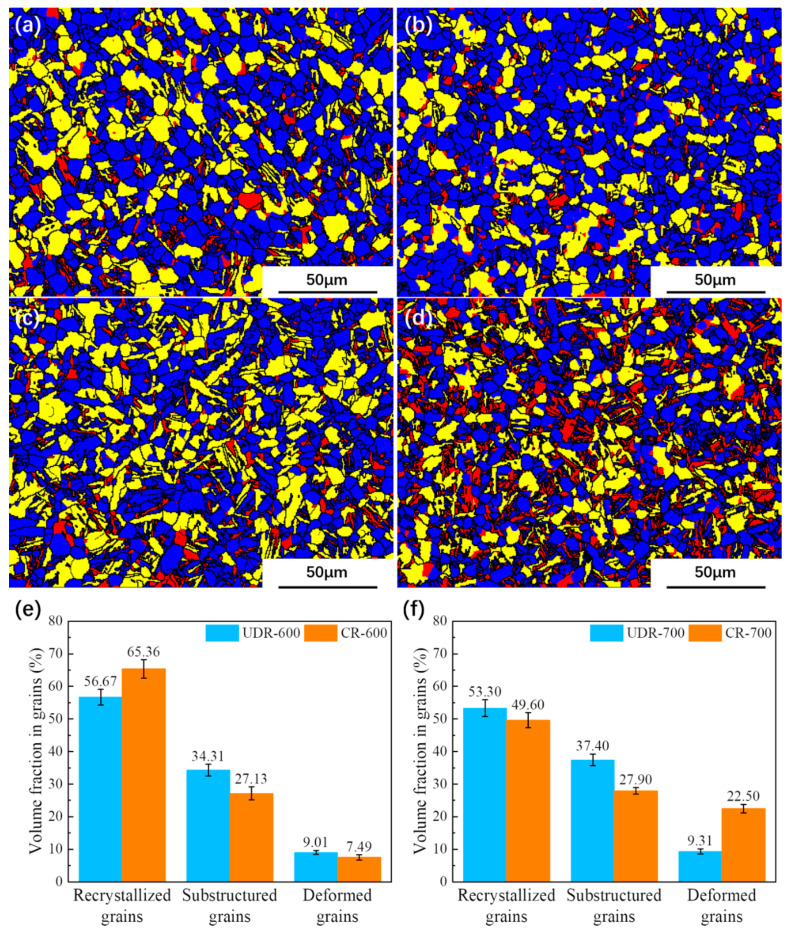
Recrystallization maps of Ti65 alloy sheets: (**a**) UDR-600; (**b**) CR-600; (**c**) UDR-700; and (**d**) CR-700. Recrystallized grains are marked as blue, substructure grains as yellow and deformed grains as red. Recrystallization diagrams of (**e**) 600 °C samples and (**f**) 700 °C samples.

In Figure 8, grains with maximum size are marked as red, and grains with minimum size as blue. It can be seen that there are more fine grains in the UDR-600 sample, which is in line with what observed in Figure 6a. Compared to 600 °C samples, more coarse grains are observed in 700 °C samples, but finer grains in the UDR-700 sample, too. Figure 9 shows recrystallization maps and diagrams. Recrystallized grains are marked as blue, substructured grains as yellow, and deformed grains as red. For all, recrystallized grains dominant, and the fraction of recrystallized grains in the UDR-600 sample is 56.67%. In general, the UDR sample has more stored energy and hence high driving force for recrystallization, which leads to higher fraction of recrystallized grain [22]. However, there is an abnormally high extent recrystallization in the CR-600 sample with the fraction of recrystallized grains in 65.36%, which is contrary to previous research on other alloys [33]. The reason may be attributed to dynamic recrystallization (DRX) during hot rolling process. The mechanism of DRX consists of discontinuous dynamic recrystallization (DDRX) and continuous dynamic recrystallization (CDRX). In general, the CDRX process plays a dominant role at a low deformation temperature (990 °C applied in the present work), and it is carried out by cross-slip of dislocations on non-basal planes [34]. More active slip systems during CR process of Ti65 alloy promote the CDRX proceed, and hence high recrystallization fraction of the CR-600 sample. The fraction of recrystallized grains in the UDR-700 sample decreases to 53.30%, which may be attributed to not enough grains for statistic or completed recrystallization. It is more than that in the CR-700 sample with the fraction of 49.6%. Another thing to note is that the α_s_ phase in the CR-600 sample is small and densely distributed. In the current conventional EBSD scanning system, it is difficult to accurately calibrate such small and densely distributed α_s_, and it is very easy to mark them as large equiaxed α grains. This may also be one of the reasons why the marked recrystallized grains are more. The αs phase in the CR-700 sample is relatively coarser, so it is easier to mark it systematically. However, during the β↔α phase transformation, significant lattice distortion occurs, which is often mislabeled as deformed grains in the analysis and statistical work of EBSD.

**Figure 10:**
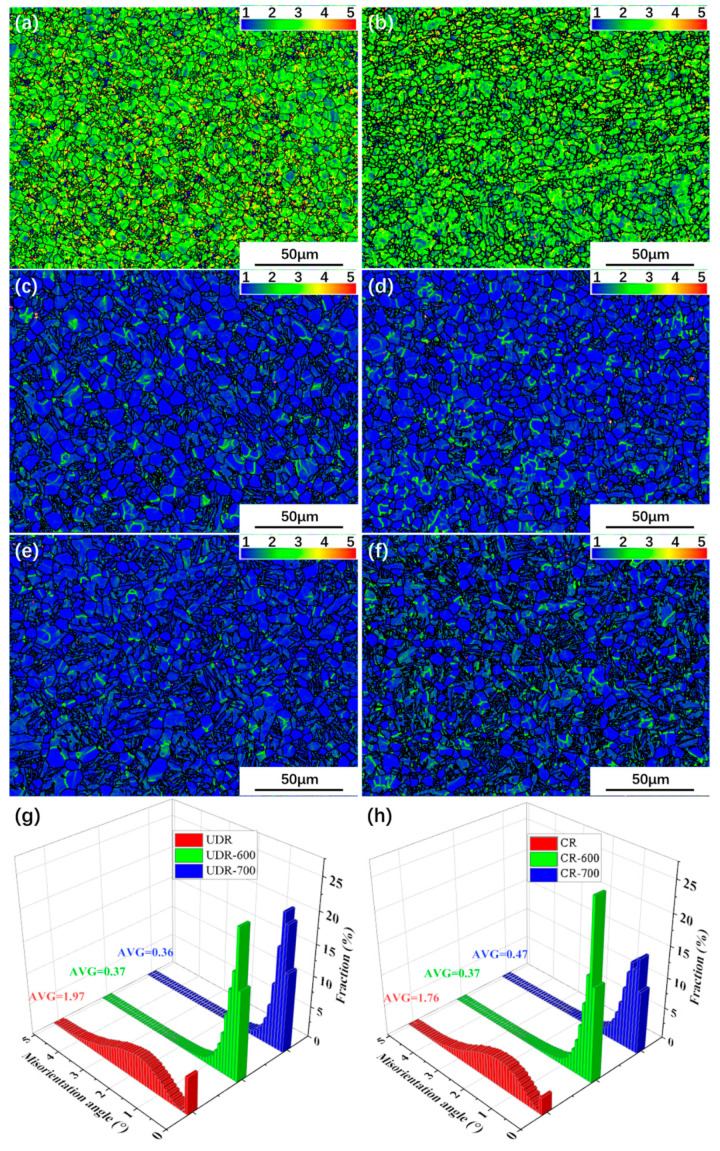
Kernel Average Misorientation (KAM) maps of Ti65 alloy sheets: (**a**) UDR; (**b**) CR; (**c**) UDR-600; (**d**) CR-600; (**e**) UDR-700; and (**f**) CR-700. The regions with high KAM are colored as red, while regions with low KAM as blue. KAM histograms of (**g**) UDR samples and (**h**) CR samples.

The KAM maps obtained by calculating the average orientation differences between adjacent points can show the average local misorientation below the subgrain angle, and hence represent and locate deformed region and dislocation density [7] Regions with high KAM are colored as red, and regions with low KAM as blue. Due to high strain, KAM maps of the UDR sample and the CR sample, as shown in Figure 10a,b, both are full of green regions, with lots of yellow or even red regions, which demonstrates high density dislocation, but the average KAM value of the CR sample (1.76) is less than the UDR sample (1.97). Due to more active slip system, there is less dislocation accumulation in the CR sample, and hence with low dislocation density. The average KAM value of the UDR-600 sample is the same as the CR-600 sample, with number of 0.37, which means that it is more effective for UDR samples to decrease dislocation density during the same heat treatment, which is attributed to more stored energy in the UDR sample. The average KAM value of the UDR-700 sample is nearly the same as the UDR-600 sample, but it rises from 0.37 in the CR-600 sample to 0.47 in the CR-700 sample, which is attributed to the decrease of the fraction of recrystallized grains.

### 3.2. α Phase Texture Characterization of the Ti65 Alloy Sheets

**Figure 11:**
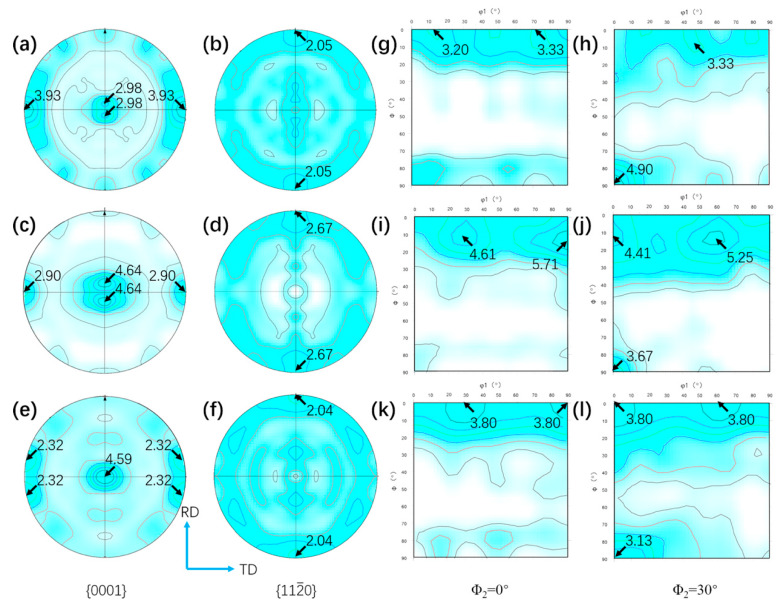
{0001} and $\left\{11\bar{2}0\right\}$ pole figures and ODF maps of UDR Ti65 alloy sheets: (**a**,**b**,**g**,**h**) UDR; (**c**,**d**,**i**,**j**) UDR-600; and (**e**,**f**,**k**,**l**) UDR-700.

**Figure 12:**
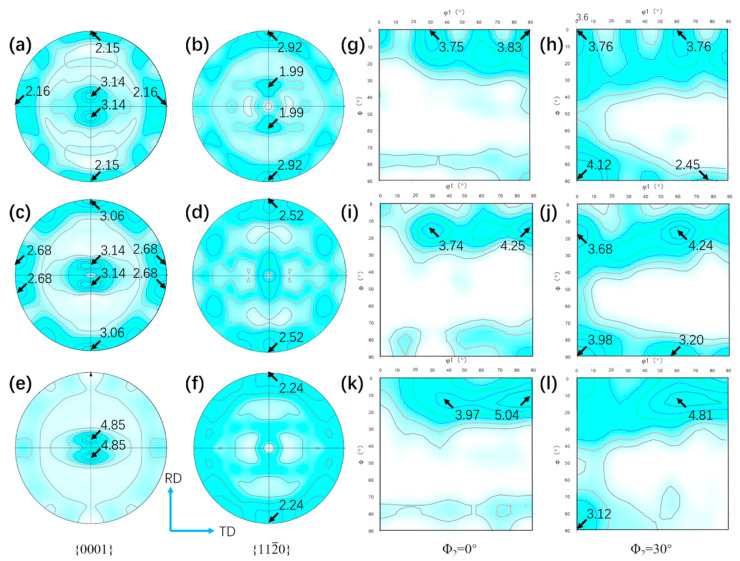
{0001} and $\left\{11\bar{2}0\right\}$ pole figures and ODF maps of CR Ti65 alloy sheets: (**a**,**b**,**g**,**h**) CR; (**c**,**d**,**i**,**j**) CR-600; and (**e**,**f**,**k**,**l**) CR-700.

**Figure 13:**
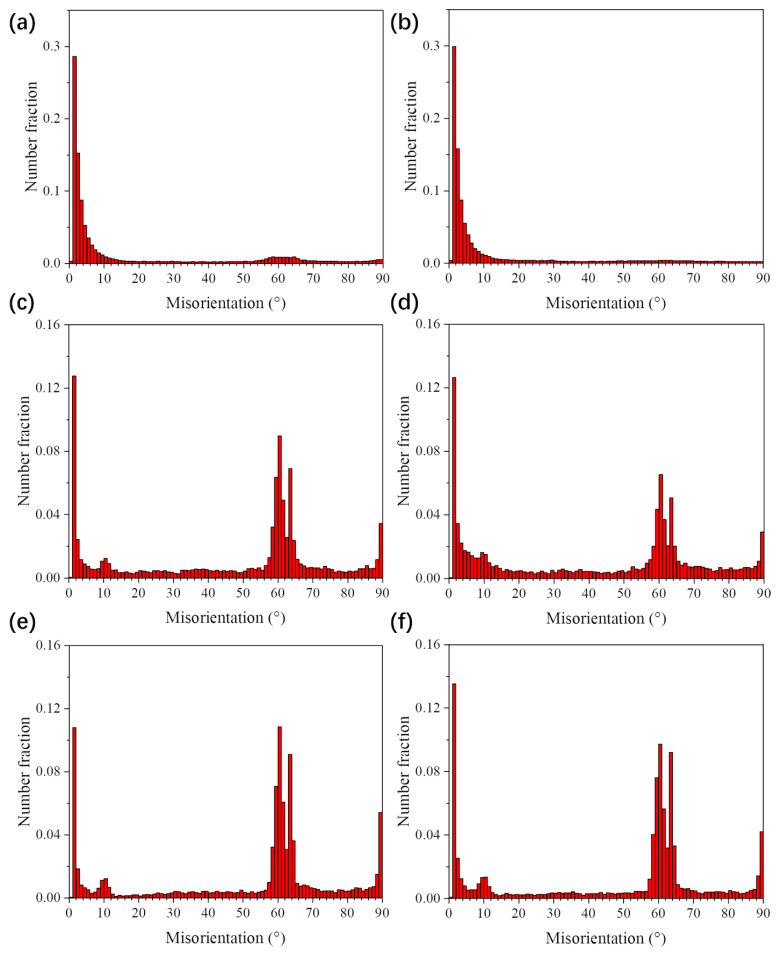
Distribution of α grain boundary misorientation of Ti65 alloy sheets: (**a**) UDR; (**b**) CR; (**c**) UDR-600; (**d**) CR-600; (**e**) UDR-700; and (**f**) CR-700.

Figure 11a–f present {0001} and $\left\{11\bar{2}0\right\}$ pole figures of UDR samples, and Figure 12a–f for CR samples. Figure 11g–l and Figure 12g–l show ODF maps of UDR and CR samples, respectively. B-type texture with intensity of 2.98 and strong T-type texture with intensity of 3.93 of the UDR sample are observed in Figure 11a, while no obvious texture is observed from $\left\{11\bar{2}0\right\}$ pole figure. The main texture components measured from ODF maps are {0001} basal fiber and ($01\bar{1}0$)<$2\bar{1}\bar{1}0$>. Distribution of α grain boundary misorientation for samples in Figure 13 shows the fraction of LAGB in UDR sample is higher than 50%, indicating concentration of grain orientation, and it becomes slightly higher in the CR sample, which may be attributed to profuse accumulation of dislocation caused by high slip activation during the CR process [35]. As can be conferred from Figure 12a, besides B-type texture with intensity of 3.14 and T-type texture with low intensity of 2.16, there is a new texture with {0001} pole concentrated around RD denoted as R-type texture, which corresponds to the B/T-type texture. Its intensity is 2.15, and main texture component is ($01\bar{1}0$)<0001>. For the UDR-600 sample, intensity of B-type texture rises to 4.64, while intensity of T-type decreases to 2.90. With rising heat treatment temperature, nearly no change in B-type texture with intensity of 4.59 is observed at the UDR-700 sample, while T-type texture is weakened with c-axis tilted from TD to RD and intensity of 2.32. Intensity of B-type texture rises from 3.14 in the CR sample to 4.85 in the CR-700 sample, while both T/R-type texture are dramatically weakened or even disappear, indicating evolution of texture in CR samples is more obvious, and it can be conferred that high temperature aging treatment can effectively weaken anisotropy of mechanical properties as a result of the strong B-type texture. However, the texture evolution of the CR-600 sample is different from others, as the intensity of B-type texture keeps constant and that of T/R-type texture does not decrease but increase, which is related to the abnormal high extent recrystallization in the CR-600 sample. The fraction of LAGB for both UDR and CR samples decreases significantly after solution plus aging treatment, and shows spread of texture after heat treatment. As shown in Figure 13c–f, the fraction of grain boundaries with misorientation of 60° and 90° increases dramatically from nearly 0%, and higher the aging temperature is, higher the fraction of grain boundaries with misorientation of 60° and 90° is. According to the Burgers orientation relationship, misorientation of c-axis of 12 α variants should be 60° or 90°, and results in scattered orientations and relatively weak texture. It can be conferred that part of the original texture becomes weaker with increasing aging temperature, and the variant selection during phase transformation process may be one of the main reasons for it.

### 3.3. The Differences in Mechanical Properties Caused by Rolling Mode

The tensile properties of UDR sheet and CR sheet of Ti65 alloy are given in Table 2. It is seen that the average values of YS and TS of the UDR sheet are slightly higher than the CR sheet, whether along RD direction or TD direction, but the differences of YS/TS between RD and TD specimens in the CR sheet are lower than the UDR sheet. The average values of EL of the UDR sheet along RD and TD direction are 7.7% and 5.3%, respectively, which is higher than that in the CR sheet, with the value of 4.5% along RD direction and 4.0% along TD direction.

As mentioned, the UDR sheet has more fine grains, which results in higher strength due to fine grain strengthening effect. The high value of EL in the UDR sheet may be attributed to small grain size of equiaxed $\alpha_{p}$ and lamellar $\alpha_{s}$. The Smaller the grain size of equiaxed $\alpha_{p}$ and lamellar $\alpha_{s}$ is, the smaller the effective slip length is, which leads to higher elongation [36]. According to the Schmid factor, the activity of slip of grains belongs to texture that is harder along the direction that is close or even parallel to the orientation of texture, and hence higher strength but lower elongation [37]. With T/R-type texture, there is low difference between the changes in tensile properties resulted by texture along RD and TD direction in the CR sheet, which means low anisotropy of tensile properties. In summary, the sheet rolled by CR process has slightly lower tensile properties but much lower anisotropy of tensile properties.

## 4. Discussion

### 4.1. Role of Rolling Mode on Deformation Texture

In general, three slip systems and one twining system are the principal modes during deformation of HCP structure crystal [6]: (1) basal $\left\{0001\right\}<11\bar{2}0>$; (2) prismatic $\left\{10\bar{1}0\right\}<11\bar{2}0>$; (3) pyramidal $\left\{11\bar{2}2\right\}<11\bar{2}\bar{3}>$; and $\left\{10\bar{1}1\right\}<11\bar{2}0>$; and (4) twinning $\left\{10\bar{1}0\right\}$ slip on $\left\{10\bar{1}2\right\}$. Based on the ratio of c/a and temperature of thermal deformation process, there are differences in the principal slip systems for HCP structure crystals. With low ratio of c/a of Ti (1.587), the ratio of value of critical resolved shear stress (CRSS) of basal: prismatic: pyramidal slip systems is 1:0.7:3 [38]. Hence, the principal slip systems of Ti is prismatic $\left\{10\bar{1}0\right\}<11\bar{2}0>$ and the secondary is basal $\left\{0001\right\}<11\bar{2}0>$, while pyramidal slip system can hardly be active. However, as difference between basal and prismatic slip system decreases with increasing temperature, it can be considered that difficulty of activity of basal and prismatic slip system is nearly equal when temperature reaches a certain value or even greater than. As a result, evolution of microstructure and texture during hot rolling are actually governed by the combined effect of activity of prismatic $\left\{10\bar{1}0\right\}<11\bar{2}0>$ and basal $\left\{0001\right\}<11\bar{2}0>$ slip systems.

During the UDR process, the sheet was subjected to tensile forces along RD, resulting in rotation of grains by the activity of prismatic $\left\{10\bar{1}0\right\}<11\bar{2}0>$ and basal $\left\{0001\right\}<11\bar{2}0>$ slip systems. Therefore, textures with basal poles titled away from the normal direction toward the transverse direction formed. However, with a different strain path, it varied during the CR process. Because of the rotation of 90° applied to the sheet during CR process, direction of tensile stress to grains in the CR sheet changed, in turn leading to changes of Schmid factor for orientations, which eventually results in hardening of initially soft prismatic slip systems. Hence, basal slip system became the principal slip system [39]. Besides, slip systems which were inactive along RD during UDR process got activated along TD during the CR process [40]. With the combination of basal, harden prismatic, and other new slip systems, there were more elongated grains in the CR sample (Figure 5a,b), and R-type texture formed besides B-type and T-type texture (Figure 12a).

### 4.2. Effect of Heat Treatment on Evolution of Microstructure and Texture

Recrystallization, spheroidization, and phase transformation happened during the heat treatment process. Stored energy of adjacent deformed grains and grain boundary energy are the main factors during the recrystallization process, and hence nucleation occurs preferentially where high stored energy exists. Few elongated grains are observed in 600 °C and 700 °C samples, which indicates that the recrystallized grains have nucleated and grown. With low heat treatment temperature, low thermal activation energy for recrystallization process results in the low rate of nucleation of recrystallized grains, and hence, growth is delayed. When temperature increases, the rate of nucleation increases rapidly until reaching the maximum, which leads to rapid exhaustion of stored energy of adjacent deformed grains. Hence the recrystallization process is dominated by growth of grains. As larger grains grow by consuming smaller grains, there is a reduction of grain boundary energy [22]. In general, with less slip system, stored energy of the UDR sample is higher. With the same heat treatment temperature, there is more nucleation in UDR samples, while growth of grains is delayed, resulting in higher volume fraction of recrystallized grains (Figure 9) but finer grains (Figure 8), which is exactly opposite in CR samples. As growth of secondary texture is restricted by orientation pinning effect [41] of principal texture, the secondary texture consumes small grains with high misorientation with the matrix and the component of the principal texture, leading to the growth of the secondary texture. As the principal texture of the UDR sample is T-type texture while the secondary one is B-type texture, the intensity of B-type texture increases while T-type texture is weakened (Figure 11). But for the CR sample, due to existence of two minor texture, i.e., T-type texture and R-type texture, orientation pinning effect is weakened, which may lead to less increase for both T-type and R-type texture after recrystallization (Figure 12). However, with the abnormal increase of volume fraction of recrystallized grains in the CR-600 sample, the abnormal increase of T/R-type texture is observed (Figure 12c). Due to drastic reduction of recrystallized grains in the CR-700 sample (Figure 9), the strengthening of orientation pinning effect to T/R-type texture is significantly weakened, which is one of the reasons for disappearance of T/R-type texture in the CR-700 sample.

Contrary to the opinions that orientations of grains change nothing after spheroidization, with the study to the role of orientation dependent spheroidization in Ti-6Al-4V alloy, Roy et al. considered that the formation of HAGB and the rotation of sub-grain inside the prior-deformed α-lamellar happened during spheroidization of $\alpha_{p}$, which leads to spread of orientation of texture [14]. In general, further deformation can facilitate the transformation from LAGB to HAGB by dislocation accumulation, along with heat treatment. As shown in Figure 13c–f, the fraction of HAGB in UDR samples is less than the fraction in CR samples in the same heat treatment, indicating weaker effect of spheroidization of $\alpha_{p}$ and hence less spread of texture. 

As mentioned, a single orientation β transforms to 12 orientations variants during the β→α process. If with equal probability, no texture appears, which is contrary to the present work. There are lots of α variant with different orientations after phase transformation if no $\alpha_{p}$ existing in β grain boundaries, while if $\alpha_{p}$ exists in, α variants are inclined to be parallel or close to the orientation of $\alpha_{p}$ without deviating from the Burgers orientation relationship, i.e., the adjacent $\alpha_{p}$ can effectively affect the orientation of α variant. It is known that $\alpha_{p}$ with high residual lattice strain is more likely to transform to β, and it can be conferred from the study of Adam [42] that prismatic {$10\bar{1}0$} and {$11\bar{2}0$} $\alpha_{p}$ orientations accumulate significantly more residual lattice strain than other orientations, resulting in most of residual $\alpha_{p}$ belonging to the component of B-type texture. Hence, due to variant selection during the phase transformation, the intensity of B-type texture significantly increases, and with increasing heat treatment temperature, more newly formed α variants lead to stronger variant selection, results in higher intensity of B-type texture. This may be the reason for the abnormal increase of T/R-type texture. Effect of variant selection to texture may be weaker than the orientation pinging effect at 600 °C, results in enhancement of T/R-type texture, while effect of variant selection is stronger at 700 °C, results in disappearance of T/R-type texture and increased intensity of B-type texture.

## 5. Conclusions

In the current study, the evolution of microstructure and texture of the new high temperature Ti65 alloy sheets developed by UDR and CR process and followed by solution and aging treatment was investigated. The following conclusions can be drawn:

(1) The microstructure of the UDR sample consists of equiaxed $\alpha_{p}$, lamellar $\alpha_{s}$, and few elongated $\alpha_{p}$. Due to a more active slip system resulting from transformed direction, the microstructure of the CR sample is composed of more elongated $\alpha_{p}$. With increasing aging temperature, both microstructures transform to duplex microstructure, and the thicknesses of lamellar $\alpha_{s}$ increases.

(2) The texture of the UDR sample is the combination of minor B-type and major T-type texture, and the main component is basal {0001} fiber texture and {$01\bar{1}0$}$<2\bar{1}\bar{1}0>$, respectively. During the CR process, the $\left\{01\bar{1}0\right\}$<0001> texture characterized as R-type texture forms besides the B/T-type texture. With increasing aging temperature, both B-type texture become stronger, while the T/R-type texture are weakened. However, an abnormal increasing of T/R-type texture with constant B-type texture happens in the CR-600 sample.

(3) Due to less active slip systems, there is higher difficulty for Ti65 alloy sheet to deform during UDR process. However, with more stored energy, heat treated UDR samples have more recrystallized grains but with smaller average grain diameter.

(4) The microstructure and texture evolution during heat treatments is resulted by the combination of recrystallization, spheroidization, and variant selection, and the abnormal increasing of T/R-type texture in the CR-600 sample is related to high recrystallization fraction.

## Figures and Tables

**Table 1:** Details of rolling modes and heat treatments in the current study.

Sample	Rolling Process at 990 °C	Heat Treatment
Unidirectional rolling (UDR)	18 mm→9 mm→4.5 mm→2 mm	-
UDR-600		990 °C/30 min AC + 600 °C/5 h AC
UDR-700		990 °C/30 min AC + 700 °C/5 h AC
Cross rolling (CR)	18 mm→9 mm→rotate 90°→4.5 mm→2 mm	-
CR-600		990 °C/30 min AC + 600 °C/5 h AC
CR-700		990 °C/30 min AC + 700 °C/5 h AC

**Table 2:** Tensile properties of Ti65 alloy sheet at room temperature. Abbreviations: YS, yield strength; TS, tensile strength; EL, elongation.

Sample	Average YS/MPa	Average TS/MPa	Average EL/MPa
UDR-RD	1070	1203	7.7
UDR-TD	1089	1186	5.3
CR-RD	1042	1177	4.5
CR-TD	1028	1179	4.0
